# Construction of the Infection Curve of Local Cases of COVID-19 in Hong Kong using Back-Projection

**DOI:** 10.3390/ijerph17186909

**Published:** 2020-09-21

**Authors:** Pui Hing Chau, Wei Ying Li, Paul S. F. Yip

**Affiliations:** 1 School of Nursing, The University of Hong Kong, Hong Kong; u3003886@connect.hku.hk; 2 Department of Social Work and Social Administration, The University of Hong Kong, Hong Kong; sfpyip@hku.hk; 3 Hong Kong Jockey Club Centre for Suicide Research and Prevention, The University of Hong Kong, Hong Kong

**Keywords:** epidemic curve, back-projection, Hong Kong, preventive measures, coronavirus disease, COVID-19

## Abstract

This study aimed to estimate the infection curve of local cases of the coronavirus disease (COVID-19) in Hong Kong and identify major events and preventive measures associated with the trajectory of the infection curve in the first two waves. The daily number of onset local cases was used to estimate the daily number of infections based on back-projection. The estimated infection curve was examined to identify the preventive measures or major events associated with its trajectory. Until 30 April 2020, there were 422 confirmed local cases. The infection curve of the local cases in Hong Kong was constructed and used for evaluating the impacts of various policies and events in a narrative manner. Social gatherings and some pre-implementation announcements on inbound traveler policies coincided with peaks on the infection curve.

## 1. Introduction

The World Health Organization (WHO) declared the coronavirus disease (COVID-19) as a pandemic [1]. It has been demonstrated that social distancing, contact tracing, patient isolation, early diagnosis, city lockdown, and travel restrictions were effective measures in slowing down the spread of COVID-19 [2,3,4,5,6,7,8,9,10,11,12,13]. For the public to be prepared for dramatic changes, both physical and mental, a pre-implementation announcement is usually made. Depending on the actual time of the announcement, the time gap ranges from less than 24 h to a couple of days. To date, the impact of this window of infection and its unintended consequences have not been investigated.

Apart from using simulations under the Susceptible-Exposed-Infectious-Removed (SEIR) model, an examination of the trajectory of the epidemic curve provides some insight into the impacts of various policies and events in a narrative manner. With one exception [14], to date, those investigations on the effectiveness of preventive measures were based on epidemic curves mainly constructed using the date of diagnosis or onset [2,6,8,9,10,11,12,13]. The estimated mean incubation period of the COVID-19 varies from 4.9 to 7.4 days across studies [15]. The epidemic curve based on the infection date, however, would be more accurate to assess the effectiveness of various measures and the impacts of the events. As the date of infection is unobservable, it has to be estimated. The back-projection technique was originally developed to construct the infection curve for the human immunodeficiency virus (HIV)/acquired immune deficiency syndrome (AIDS) epidemic [16,17]. In 2003, the authors applied the back-projection method to the severe acute respiratory syndrome (SARS) epidemic [18]. The current study constructed an epidemic curve based on the date of infection estimated by the back-projection method to minimize the effects of the incubation time and reflect the possible impacts of various measures and events better.

Up until 31 August 2020, Hong Kong had 4811 confirmed COVID-19 cases with 89 deaths. From January to April 2020, most of the cases were imported and clustered with known sources, and were considered as the first two waves of the infection. The third wave started in July, still continuing at the time of this publication. Most of those cases were local, and the sources of many of which were unknown. The present study focused on the first two waves of local infections in Hong Kong. All the government announcements were systematically recorded on the official website, thus providing reliable information on the pre-implementation announcement dates and the implementation dates of the preventive measures. This provided a trusted source to examine the possible association between the pre-implementation announcements and the infection curve in a narrative manner. Our objective was to estimate the infection curve of the local cases of the first two waves of COVID-19 in Hong Kong using the back-projection method and explore the effectiveness of the preventive measures, including the possible impacts of the pre-implementation announcements by the local government.

## 2. Materials and Methods

The daily number of confirmed cases of COVID-19 reported by 30 April 2020 was obtained from the website of the Centre for Health Protection, Hong Kong (https://www.chp.gov.hk/). A confirmed case was defined as a person with laboratory confirmation of COVID-19 infection, irrespective of clinical signs and symptoms [19]. As the date of infection of the imported cases cannot reflect the impact of the preventive measures implemented locally, all imported cases were excluded from the analysis. Until 30 April 2020, there were 1038 confirmed cases in Hong Kong, of which 422 were local cases and included in this study. Among the 422 cases, 46 were asymptomatic and the date of onset was proxied by the date of report in the primary analysis. A sensitivity analysis was performed by excluding the asymptomatic cases.

**Figure 1:**
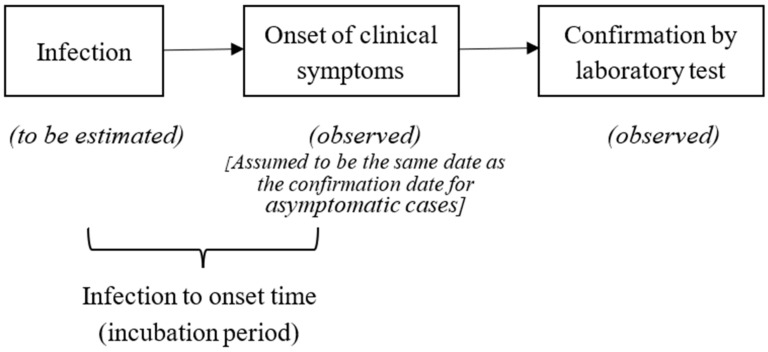
Temporal relation between infection, onset, and confirmed cases.

An infected person goes through an incubation period before the onset of the symptoms. The time when the symptoms appear is the onset time. The term “onset cases” was used to refer to the cases with onset of the symptoms on a particular day. There was also a delay between the onset of symptom(s) and the confirmation of cases. Figure 1 shows the temporal relation between infection, onset, and confirmed cases.

In this study, daily onset cases were used for the back-projection method. Let *t =* 1, 2,…, *τ* denote the days, where *t* = 1 denotes 12 January 2020. As the earliest onset date was 22 January 2020, it was assumed that there was no infection 10 days prior to that date. Furthermore, this was consistent with the fact that the earliest arrival date of imported cases was 14 January 2020. As the last time point was 30 April 2020, *τ* had been set to 110. The daily mean number of onset COVID-19 cases (*µ_t_*) was expressed in terms of the daily mean number of infection (*λ_s_*, *s*=1,…, *t*) by the convolution equation:

(1) $$\mu_{t}=\sum_{s=1}^{t}\lambda_{s}f_{t-s,s}$$

where *f_t-s,s_* is the probability function of the incubation period, that is, the probability that an individual infected at time *s* developed a symptom after a period of length *t-s*. In other words, infected cases on day 1 went through an incubation period of *t-1* days with the probability specified by the probability function of the incubation period and had onset of symptoms on day *t;* then infected cases on day 2 went through an incubation period of *t-2* days; infected cases on day 3 went through an incubation period of *t-3* days and so on, up to infected cases on day *t* that went through an incubation period of 0 days. The sum of all these cases was the total number of onset cases on day *t*. Based on a recent meta-analysis on the incubation period established from eight studies [15], the probability function of the incubation period of COVID-19 was taken as a log-normal distribution with scale and shape parameters of 1.63 and 0.5, respectively. This corresponded to a mean of 5.8 days, a median of 5.1 days, and a 95th percentile of 11.7 days. The EMS (Estimate-Maximize-Smooth) algorithm was used to estimate the daily number of infection and pointwise 95% confidence interval was constructed by the bootstrap procedure. Details of the back-projection method are reported elsewhere [16,17]. Python was used for running the algorithm [20].

The estimated infection curve was closely examined in relation to the major events and policies announced and/or implemented by the local government. The major events and policies were obtained from the official websites (www.info.gov.hk/gia/ and www.news.gov.hk). Table S1 shows the selected government policies and major events in the period under examination.

## 3. Results

### 3.1. Estimated Infection Curve

**Figure 2:**
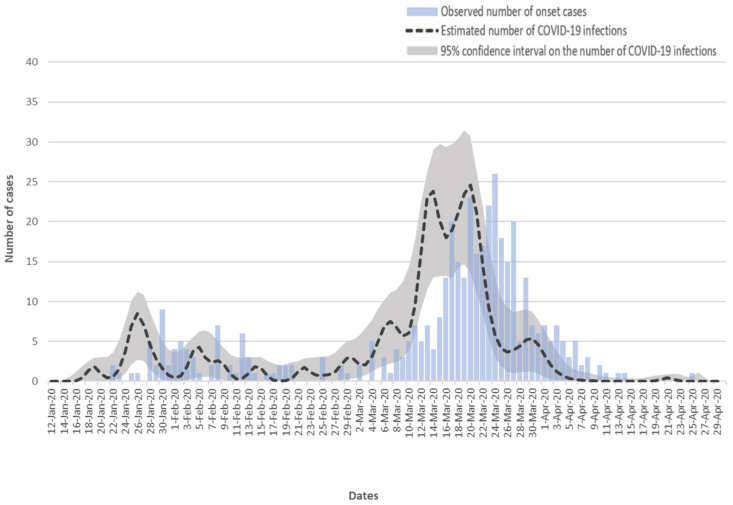
Estimated daily number of infections of the coronavirus disease (COVID-19) with pointwise 95% confidence intervals and the observed daily number of onset cases in Hong Kong from 12 January to 30 April 2020.

The number of COVID-19 infections over this period was estimated to be 422. Figure 2 gives the estimated daily number of COVID-19 infections, the pointwise 95% confidence intervals on the estimated infection number, and the observed daily number of onset cases for the period from 12 January 2020 to 30 April 2020. It was estimated that the first infection occurred around 18 January 2020, roughly four days after the arrival of the first imported case on 14 January 2020. The highest peak was estimated around mid-March 2020, which was in the middle of the second wave of infection which started in March. Zero infection was estimated since 4 April 2020.

### 3.2. Possible Association with Social Gathering

In the first wave, the estimated infected cases in late January 2020 could be mainly attributable to the social and family gatherings around the Chinese New Year. It was reported from contact history tracing that 6 infected people attended a family dinner in North Point on 26 January 2020, 11 infected people attended a family gathering at a party room in Kwun Tong on 26 January 2020, and 13 infected people visited a Buddhist worship hall in North Point between 25 January and early February 2020.

In the second wave, it was reported that there were two large-scale gatherings on 14 March 2020, one involved 80 guests at a wedding party and the other involved over 100 guests at a private party. A total of 14 confirmed cases were reported from those two gatherings. This was consistent with the peak in the infection curve on 14 March 2020. Furthermore, 72 staff and customers of some pubs and bar areas were infected, and 31 more confirmed cases had epidemiological link to these cases. The onset date of this infection cluster was reported to be from 10 March to 13 April 2020, implying infection from around early March to early April. On 24 March 2020, it was reported that 7 people went to a karaoke and all were infected.

### 3.3. Possible Association with Prevention Policies

On 26 January 2020, an announcement was made to ban inbound travelers who had visited the Hubei Province in the past 14 days prior to its actual implementation at midnight on 27 January 2020. From 25 to 29 January 2020, numerous policies were announced and implemented, including activation of the Emergency Response Level, cancellation of large-scale events, quarantine of close contacts of confirmed cases, health advice to residents returning from the Hubei province and other parts of China, suspension of non-emergent government services, closure of public facilities, home office arrangement for civil servants, and substantial reduction of traffic between Mainland China and Hong Kong. As the peak of the first wave around 24–28 January 2020 was dominated by cluster infections during social gathering, it was difficult to observe potential influence from these policies.

The announcement on 28 February 2020 about mandatory quarantine for inbound travelers who had been to Emilia-Romagna, Lombardy, or the Veneto regions in Italy or Iran in the past 14 days coincided with a local maximum. The curve, however, slightly went down after implementation of the policy on 1 March 2020. There was also an announcement on 6 March 2020 about the health declaration requirement on all inbound travelers effective from 8 March 2020. The curve declined slightly forthwith.

The peaks on 13–15 March and 18–21 March 2020 were the key features of the second wave. During 2–22 March 2020, some non-emergent government services and public facilities resumed, and civil servants returned to work in the office. Various policies on inbound travelers were announced and implemented during that period. However, their associations with the infection curve might be masked by the cluster infections in the wedding party, private party and the pubs and bar areas during the same period. On 10 March 2020, the government announced mandatory quarantine on inbound travelers from the whole of Italy, France (Bourgogne-Franche-Comte and Grand Est), Germany (North Rhine-Westphalia), Japan (Hokkaido), and Spain (La Rioja, Madrid, and Pais Vasco). This announcement coincided with the rise on the infection curve. On 13 March 2020, the government further announced mandatory quarantine on inbound travelers who had visited the Schengen Area countries. Moreover, on 15 March 2020, mandatory quarantine was announced on all inbound travelers who had traveled to Ireland, the United Kingdom, the United States, and Egypt. Then, on 17 March 2020, mandatory quarantine was announced for all inbound travelers from all overseas places, which activated the downward trend again. Starting from 20 March 2020, inbound travelers with symptoms of upper respiratory symptoms were tested at designated test centers and they had to wait for the results. For the second time, non-emergent government services and public facilities were closed from 23 March 2020, and the civil servants resumed to home office arrangement again. After implementation of these policies, the infection curve declined.

Against the declining trend, the infection number rebounded on 30 March 2020. However, the increase appeared to be not related to the pre-implementation announcements on 27 March 2020, which involved (i) strict operational instructions on the catering business (limiting the number of seats to half of the capacity, minimum distance of one and a half meters between tables, and a maximum of four seats per table); (ii) closure of scheduled premises (including amusement game centers, bathhouses, fitness centers, places of amusement, places of public entertainment, and party rooms); and (iii) ban of groups over four people in public places. The first two policies were implemented on 28 March 2020 and the third policy was implemented on 29 March 2020. Furthermore, closure of karaoke, mahjong-tin kau, and nightclub establishments was announced on 1 April 2020 and closure of bars and pubs was also announced on 2 April 2020, and implemented on 1 and 3 April 2020, respectively. After implementation of all those policies, the infection number continued to fall to zero by 4 April 2020.

### 3.4. Sensitivity Analysis

The sensitivity analysis that excluded the asymptomatic cases (Figure S1) gave a similar trajectory of the infection curve. It was noted that the local maxima from 28 February to 1 March 2020 and from 29 March to 3 April 2020 were not observed in the sensitivity analysis that excluded the asymptomatic cases. The peak on 19 March 2020 in the sensitivity analysis was lower than that which included the asymptomatic cases. It might be that those infected in those periods were mostly asymptomatic.

## 4. Discussion

The current study constructed the infection curve of local confirmed COVID-19 cases in Hong Kong. From studying the estimated curve closely with the major events and preventive measures, it was observed that a strict mandatory quarantine order on inbound travelers coincided with the decline from the peak in the infection number. However, some pre-implementation announcements of such policies and social gatherings corresponded with sharp increases in the infected cases. The closure of public facilities and home office arrangement might have helped to slow down the spread.

It is common practice around the globe to pre-announce government policies. In Hong Kong, the press conference was usually held at 4:30 pm. For policies to be implemented the following day, such an announcement would only leave several hours before the actual implementation. To stir up reactions, such a short period may not be sufficient. For transportation from overseas countries to Hong Kong, more time is needed for preparation. Thus, the announcements of mandatory quarantine on people returning from overseas were made over 24 h prior to implementation. Instead of using the time to well plan for the 14-day quarantine, those from overseas, however, rushed back to Hong Kong to avoid the mandatory quarantine. It was reported that 46 confirmed imported cases (the largest daily imported cases) entered Hong Kong on 18 March 2020, just before implementation of the mandatory quarantine policy. To make things worse, social gathering still went on during that period when cluster infections related to some bars and pub areas were reported. It is understandable though for the government to announce the policies in advance to allow the people to be better prepared. However, in Hong Kong, many residents rushed back before the implementation to avoid the mandatory quarantine, thus increasing the risk of transmission in the community. To minimize the opportunity of infection, pre-implementation announcements should be made as close to the implementation date as possible. Meanwhile, comprehensive support plans for those being quarantined and groundwork should simultaneously be in place.

Quarantines on inbound travelers appeared to be effective. According to a study in Singapore, rapid identification and isolation, quarantine of close contacts, and active monitoring of other contacts were effective measures in controlling COVID-19 [6]. Nevertheless, it should be noted that quarantine should be combined with other public health measures to achieve the best result [7]. Quarantine polices are important and overall well-being should be catered for too. During the SARS epidemic, people felt isolated and hopeless, which resulted in a rise in the suicide rate [21,22]. While controlling the spread of the epidemic, the mental well-being of the residents should also be taken care of [23]. Fortunately, with the many social media devices, social connectedness and emotional closeness can still be maintained when practicing physical distancing. It is important to ensure those quarantined do not feel left out or forgotten. Meanwhile, it appeared that screening for COVID-19 at the airport might not be effective enough in catching a significant proportion of cases [24]. However, sample testing for all inbound travelers seemed to work well in preventing the cases from entering the Hong Kong community. Since such a policy was in force together with the mandatory quarantine policy, its effect alone could not be evaluated.

The suspension of non-emergent government services, closure of public facilities, and home office arrangement for civil servants at the end of January coincided with the drop in the number of COVID-19 infections at the end of January. Moreover, resumption of these services and work at the office in early March seemed to correlate with an increase in the infection. Subsequently, the suspension of these services and home office arrangement were implemented again at the end of March. The implementation of other policies included the suspension of education and schools. However, as these policies were implemented concurrently, an evaluation on the individual effectiveness of each policy was not possible. Indeed, a review suggested that the effect of school closure was less than that of other social distancing interventions [25]. This could be true in view of an example in Taiwan where schools and offices were not closed, yet the infection could still be maintained at a low level. It has also been reported that highly effective contact tracing and case isolation were sufficient to control a new outbreak of COVID-19 [3]. In this regard, Hong Kong has been performing contact tracing exceptionally well, nonetheless, its effect also could not be assessed in the current study.

The strength of this study was the estimation of the infection curve based on the back-projection method. The limitation of this study was that the date of onset was subject to recall bias, and a proxy date of onset was assumed on 46 asymptomatic patients. A sensitivity analysis was conducted by excluding the asymptomatic cases, and the trajectory of the infection curve remained roughly the same. An assumption made on the incubation period might also affect the estimated infection curve. Nevertheless, this study utilized the pooled estimate from a meta-analysis based on eight studies [15]. This study was a narrative study, and causality cannot be assessed. In addition, if there were more than one policy or event occurring in parallel, it would be difficult to distinguish their impacts. At the same period, there were also social events such as healthcare on strike, protests for various reasons, and panic purchase of food and toilet paper, which involved the gathering of people and reduced social distancing, yet this study did not analyze their impact. Future studies might consider using the estimated infection curve to assess the effectiveness of various preventive measures and events using the quantitative approach.

## 5. Conclusion

To conclude, the infection curve of COVID-19 could be constructed by the back-projection method. The findings of this study were consistent with previous studies on the effectiveness of some preventive measures. This study suggests that social gatherings and policy announcements and implementation sometimes coincide with changes in the infection curve; however, prospective studies designed to evaluate causality are needed to further understand this observation.

## Supplementary Materials

The following are available online at https://www.mdpi.com/1660-4601/17/18/6909/s1, Figure S1: Estimated daily number of infections of the coronavirus disease (COVID-19) in Hong Kong from 12 January to 30 April 2020, including and excluding the asymptomatic cases. Table S1: Announcements and implementations of selected policies and major events in Hong Kong.
