# Supplementary material for: Construction of the Infection Curve of Local Cases of COVID-19 in Hong Kong using Back-Projection
Source: Int J Environ Res Public Health. 2020 Sep 21;17(18):6909. doi: 10.3390/ijerph17186909 (PMC7557805; doi:10.3390/ijerph17186909)

Table S1. Announcements and implementations of selected policies and major events in Hong Kong.

Major government policies/events	Dates of Announcement	Dates of Implementation
Preparedness and Response Plan launched	January 4	January 4
Health advice on personal and environmental hygiene and outbound travel	January 5	January 5
Mandatory notification of cases	January 7	January 8
Red Outbound Travel Alert issued on Iran	January 10	January 10
Port health measures and temperature screening of inbound travelers strengthened with increased frequency of random checking at all boundary control points; patients with fever and acute respiratory symptoms who had visited Wuhan within the preceding 14 days would be immediately referred to the public hospitals	January 11	January 11
Reporting criteria revised; additional temperature checks using handheld infrared thermometers on all inbound travelers via the two daily high-speed trains with stops at Wuhan; travelers who fulfilled the reporting criteria would immediately be referred to the public hospitals	January 16	January 16
Further revision of reporting criteria	January 20	January 20
Submission of a health declaration form for all inbound travelers by air from Wuhan	January 20	January 21
Further revision of reporting criteria	January 23	January 23
Submission of a health declaration form for all inbound travelers via the high-speed train	January 23	January 24
First quarantine center ready for use	January 23	January 23
Activation of Emergency Response Level; strengthening immigration control; cancellation of large-scale events; suspension of schools	January 25	January 25
**Infection cluster**: Thirteen people visited a Buddhist worship hall in North Point between January 25 and early February and another 6 confirmed cases were epidemiologically linked to these cases	--	January 25
**Infection cluster**: Twenty people attended a family dinner in North Point and 19 people attended a family gathering at a party room in Kwun Tong, and a total of 17 confirmed cases were subsequently reported	--	January 26
Ban on non-resident inbound travelers who had visited the Hubei Province in the past 14 days	January 26	January 27, 0 am
Additional health advice for inbound travelers who had visited the Hubei Province or other places in Mainland China in the past 14 days	January 28	January 28
Submission of a health declaration form by all inbound travelers from Mainland China was expanded from those who traveled via the high-speed train to those who traveled by flight	January 28	January 29, 9 am

Suspension of non-emergent government services; closure of public facilities (all sports facilities, holiday camps, museums, performance venues, and public libraries including study rooms); home office arrangement for civil servants; appeal to employers to make flexible work arrangements	January 28	January 29
Substantial reduction of traffic between Mainland China and Hong Kong; suspension of four control points; suspension of passenger services at two control points	January 28	January 30, 0 am
Suspension of four more control points	February 3	February 4
Mandatory home quarantine on all inbound travelers from Mainland China and on those who had visited Mainland China in the past 14 days	February 5	February 8
"Enhanced Laboratory Surveillance Programme" to include people aged 18 or above with fever and respiratory symptoms or mild chest infection attending the General Out-patient Clinics and Accident and Emergency Departments of the Hospital Authority	February 18	February 19
First batch of persons completed compulsory quarantine	February 22	February 22
Red Outbound Travel Alert issued on Korea	February 24	February 24
Ban on inbound non-resident travelers from Korea; mandatory quarantine at the quarantine center on inbound resident travelers who had visited Daegu and Gyeongsangbuk-do in Korea in the past 14 days; medical surveillance on inbound resident travelers who had visited other places in Korea in the past 14 days	February 24	February 25, 6 am
Red Outbound Travel Alert issued on Emilia-Romagna, Lombardy, and the Veneto regions in Italy which cover Bologna, Milan, Venice, and Verona	February 28	February 28
"Enhanced Laboratory Surveillance Programme" to include people aged below 18 and patients with fever or respiratory symptoms subject to clinical assessment of the physician in charge, and those with fever and respiratory symptoms or mild chest infection attending the General Out-patient Clinics and Accident and Emergency Departments of the Hospital Authority	--	February 28
Mandatory quarantine at quarantine centers on all inbound travelers (regardless of whether they were Hong Kong residents) who had visited Emilia-Romagna, Lombardy, or Veneto regions in Italy or Iran in the past 14 days	February 28	March 1, 0 am
Some non-emergent government services and public facilities gradually resumed; back to work at office for civil servants also gradually resumed	--	March 2–22
"Enhanced Laboratory Surveillance Programme" to cover around 10 types of viruses	March 3	March 4
Submission of health declaration form for all inbound flight passengers	March 6	March 8, 0 am
Red Outbound Travel Alert issued on the whole of Italy, France (Bourgogne-Franche-Comte and Grand Est), Germany (North Rhine-Westphalia), Japan (Hokkaido), and Spain (La Rioja, Madrid, and Pais Vasco)	March 10	March 10
Mandatory quarantine at quarantine centers on all inbound travelers (regardless of whether they were Hong Kong residents) who had visited Italy, France (Bourgogne-Franche-Comte and Grand Est), Germany (North Rhine-	March 10	March 14, 0 am

Westphalia), Japan (Hokkaido), and Spain (La Rioja, Madrid, and Pais Vasco) in the past 14 days		
Red Outbound Travel Alert issued on the Schengen Area countries; areminder of Red Outbound Travel Alert issued on Egypt	March 13	March 13
Mandatory quarantine at home on all inbound travelers (regardless of whether they were Hong Kong residents) who had visited the Schengen Area countries (except Emilia-Romagna, Lombardy, and Veneto regions in Italy) in the past 14 days	March 13	March 17, 0 am
Mandatory quarantine at quarantine centers on inbound non-resident travelers who had visited Daegu and Gyeongsangbuk-do in Korea in the past 14 days; mandatory quarantine at home on inbound non-resident travelers who had visited other places in Korea in the past 14 days (ban on entry of non-residents from Korea relieved)	March 13	March 17, 0 am
Mandatory quarantine at home on all inbound travelers (regardless of whether they were Hong Kong residents) who had visited Italy (except Emilia-Romagna, Lombardy, and Veneto regions), France (Bourgogne-Franche-Comte and Grand Est), Germany (North Rhine-Westphalia), Japan (Hokkaido), and Spain (La Rioja, Madrid, and Pais Vasco) in the past 14 days (requirement on quarantine centers relieved)	March 13	March 14, 0 am
**Infection cluster**: Over 80 people attended a cocktail wedding party in Discovery Bay and over 100 people attended a private party in Wong Chuk Hang, and a total of 14 confirmed cases were reported	--	March 14
Red Outbound Travel Alert issued on Ireland, the United Kingdom, and the United States	March 15	March 15
Mandatory home quarantine on all inbound travelers (regardless of whether they were Hong Kong residents) who had travelled to Ireland, the United Kingdom, the United States, and Egypt in the past 14 days	March 15	March 19, 0 am
**Infection cluster**: Seventy-two staff and customers of some pubs and bar areas were infected around mid-March, and 31 confirmed cases were epidemiologically linked to this cluster	--	March
Red Outbound Travel Alert issued on all overseas countries/territories	March 17	March 17
Mandatory quarantine on all inbound travelers (regardless of whether they were Hong Kong residents) who had travelled to overseas countries or territories in the past 14 days	March 17	March 19, 0 am
"Enhanced Laboratory Surveillance Programme" to cover asymptomatic inbound travelers aged 65 or above and asymptomatic travelers residing with persons aged 65 or above	March 19	March 19 afternoon
Inbound travelers with symptoms of upper respiratory symptoms tested at the AsiaWorld-Expo and North Lantau Hospital test centers and had to wait for the results	March 19	March 20
For the second time, suspension of non-emergent government services; closure of public facilities (all sports facilities, holiday camps, museums, performance	March 22	March 23

venues, and public libraries including study rooms); home office arrangement for civil servants; appeal to employers to make flexible work arrangements		
Ban on inbound non-resident travelers from overseas countries/regions by flight or via Mainland China, Macao, and Taiwan in the past 14 days; suspension of all transit services at the airport; mandatory quarantine on all inbound travelers (regardless of whether they were Hong Kong residents) from or who had stayed in Macao and Taiwan	March 23	March 25, 0 am
Request to clubs on private recreational leases to close their recreational and sports facilities, changing rooms, and children's playrooms on their premises; appeal to other private clubs and facilities in residential complexes to adopt the same measure	March 23	--
"Enhanced Laboratory Surveillance Programme" to cover asymptomatic inbound travelers arriving from the United Kingdom, other countries in Europe, and the United States	March 24	March 25
**Infection Cluster**: Seven people went to a karaoke bar and were infected	--	March 24
Strict operation requirements on the catering business (seats < half of the normal capacity, ≥1.5 m between tables or with partitions, ≤4 people at one table, must wear masks unless consuming food or drink, body temperature screening at entrance, and provision of hand sanitizers)	March 27	March 28, 6 pm
Closure of amusement game centers, bathhouses, fitness centers, places of amusement, places of public entertainment, and party rooms	March 27	March 28, 6 pm
Ban on group gatherings of more than four people in public places	March 27	March 29, 0 am
Closure of country park barbecue sites and campsites, and all outdoor sports or recreational facilities	March 27	March 28, 6 pm
Closure of karaoke, mahjong-tin kau, and nightclub establishments, and suspension of karaoke and mahjong-tin kau activities at any catering business	April 1	April 1, 6 pm
Closure of bars and pubs, including any part of a catering business premise or clubhouse that is exclusively or mainly used for the sale or supply of intoxicating liquors for consumption	April 2	April 3, 6 pm
Shortening of the operating hours of passenger clearance services at the Shenzhen Bay Port from 10 am to 8 pm	April 2	April 3
Presentation of health certification code by all cross-boundary goods vehicle drivers entering Shenzhen and presentation of proof of a negative result of a nucleic acid test conducted within the previous seven days to the Shenzhen customs officers for examination before entering Hong Kong	April 3	April 10
Shortening of the operating hours of passenger clearance services at the Hong Kong–Zhuhai–Macao Bridge in Hong Kong Port from 10 am to 8 pm, and for private cars from 6 am to 10 pm	April 4	April 5
Test centers at the AsiaWorld-Expo and North Lantau Hospital consolidated at the AsiaWorld-Expo	April 5	April 5, 8 pm

Mandatory deep throat saliva samples taken from all asymptomatic inbound travelers arriving at the airport. Mandatory quarantine at own accommodation after sample collection	April 7	April 8
Mandatory deep throat saliva samples taken from all asymptomatic inbound travelers from the United Kingdom arriving at the airport. After sample collection, they had to wait for the results at the test center	April 7	April 9
"Enhanced Laboratory Surveillance Programme" extended to inbound travelers, who had visited the Hubei Province in the past 14 days, arriving through two control points. These travelers were required to collect their deep throat saliva samples by themselves while undergoing compulsory home quarantine, and to have their family members or friends deliver the same to the collection points	April 7	April 8
Closure of beauty parlors and massage establishments	April 8	April 10, 0 am
Confinees at the quarantine centers would stay there for 10 days and be evaluated on whether they could serve the remaining four-day quarantine period at home	April 9	April 11
Mandatory deep throat saliva samples taken from all asymptomatic inbound travelers arriving on flights from the United States and other areas in Europe and had to wait for the results	April 11	April 13
The temporary COVID-19 test center at the AsiaWorld-Expo suspended from April 19 due to the declining number of inbound travelers	April 18	April 19
Mandatory testing on all inbound travelers arriving at the airport and permission to return to the community only if the test result was negative. All asymptomatic inbound travelers arriving on flights in the afternoon or at night had to stay and wait for the virus test results at the holding center and continue quarantine at their place of accommodation only if the test result was negative	April 20	April 22
Temporary closure of beaches and suspension of life-saving services at the beaches	April 25	April 25
Public services resumed	April 29	May 4

Note: The above are extracts from www.info.gov.hk/gia/ and www.news.gov.hk, please visit the sites for a full list.

Figure S1. Estimated daily number of infections of the coronavirus disease (COVID-19) in Hong Kong from 12 January to 30 April 2020, including and excluding the asymptomatic cases.

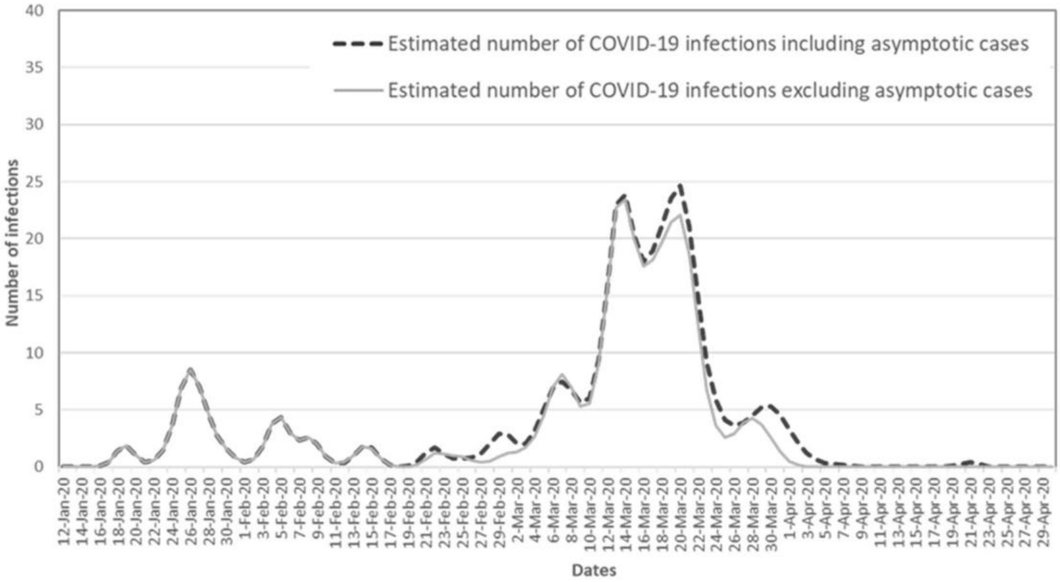

Supplement: Supplementary file 1 [file ijerph-17-06909-s001.pdf]
